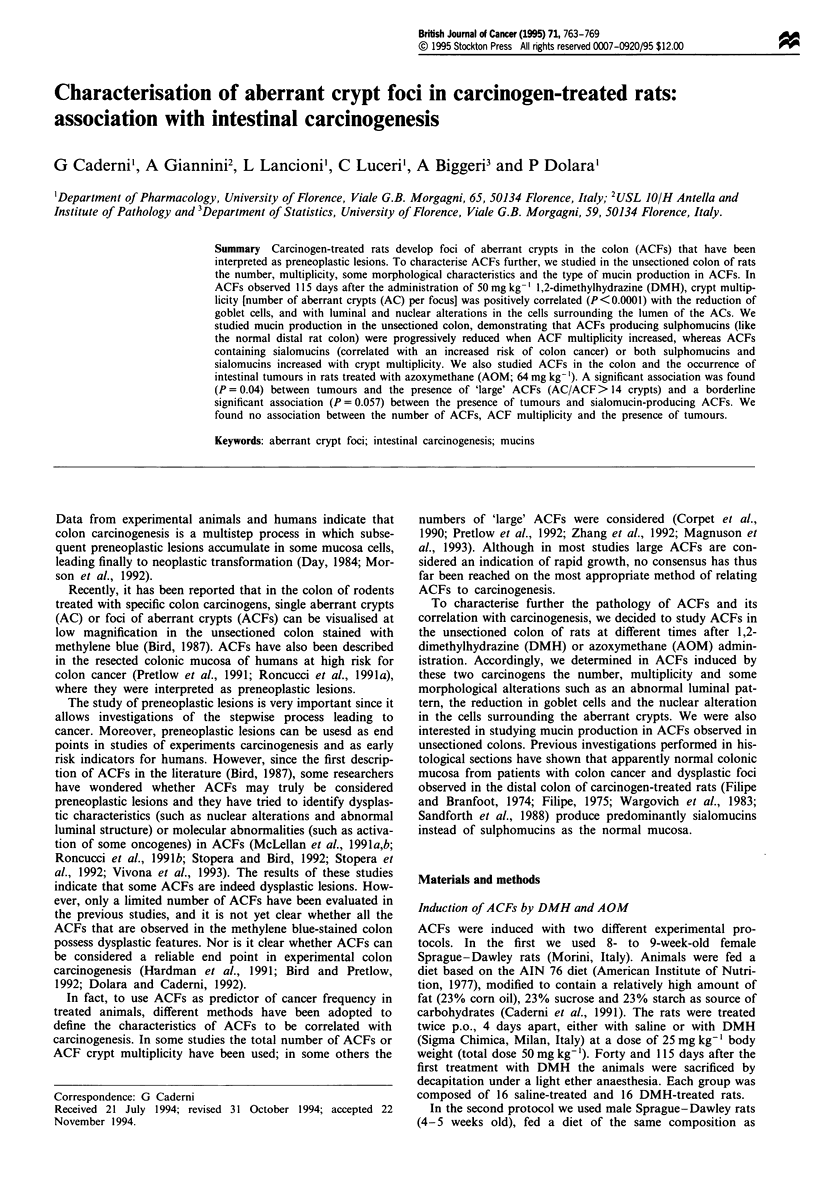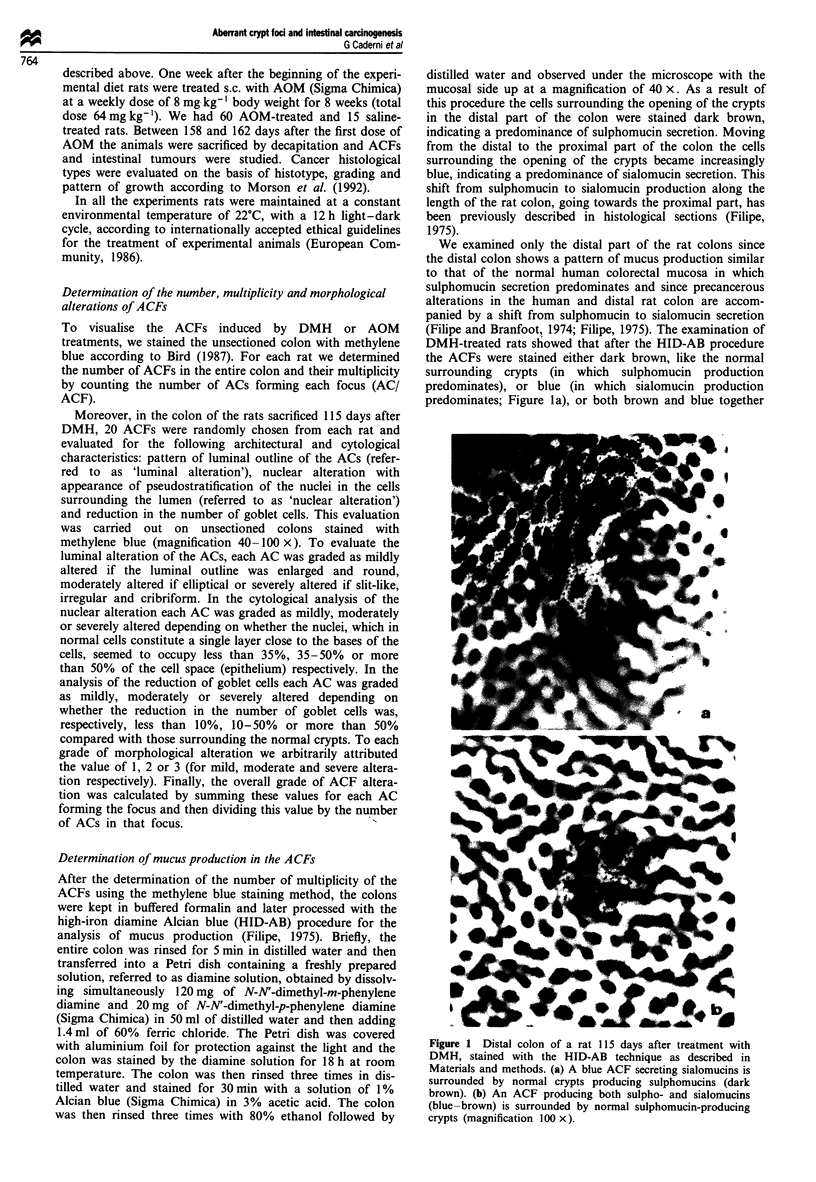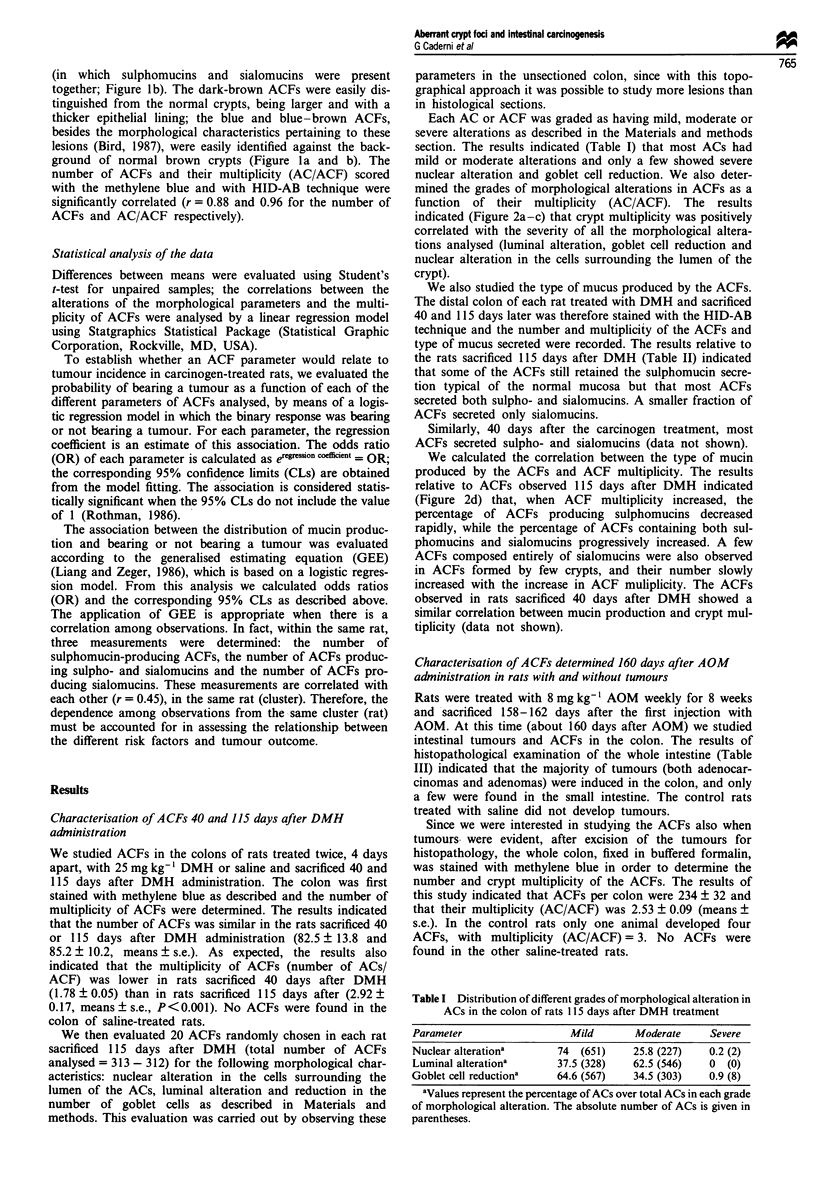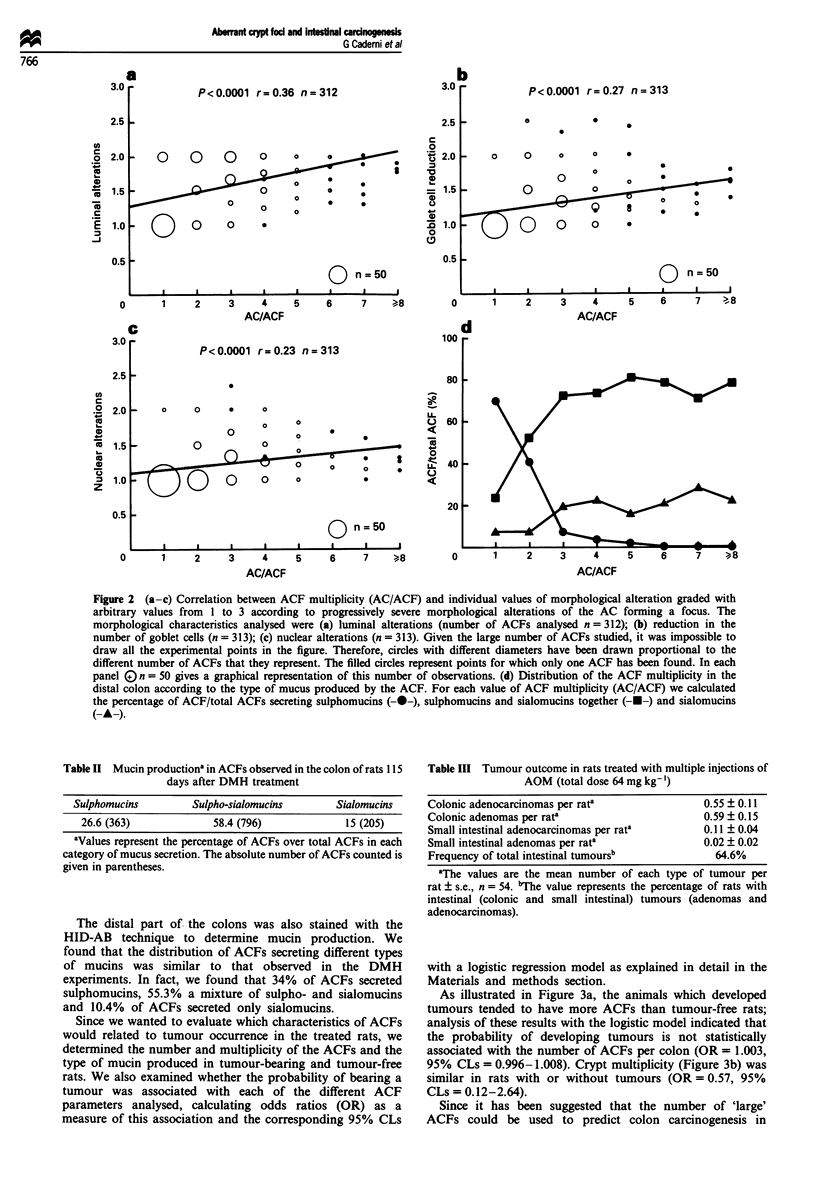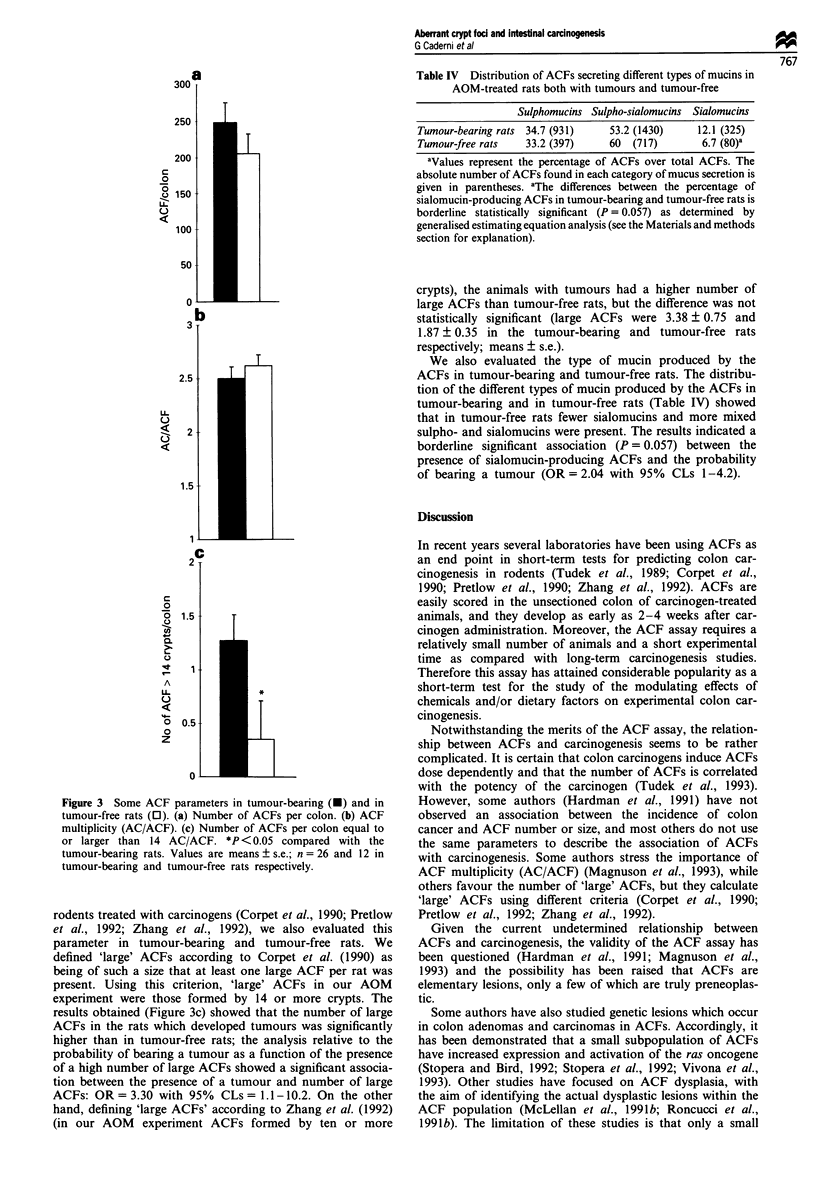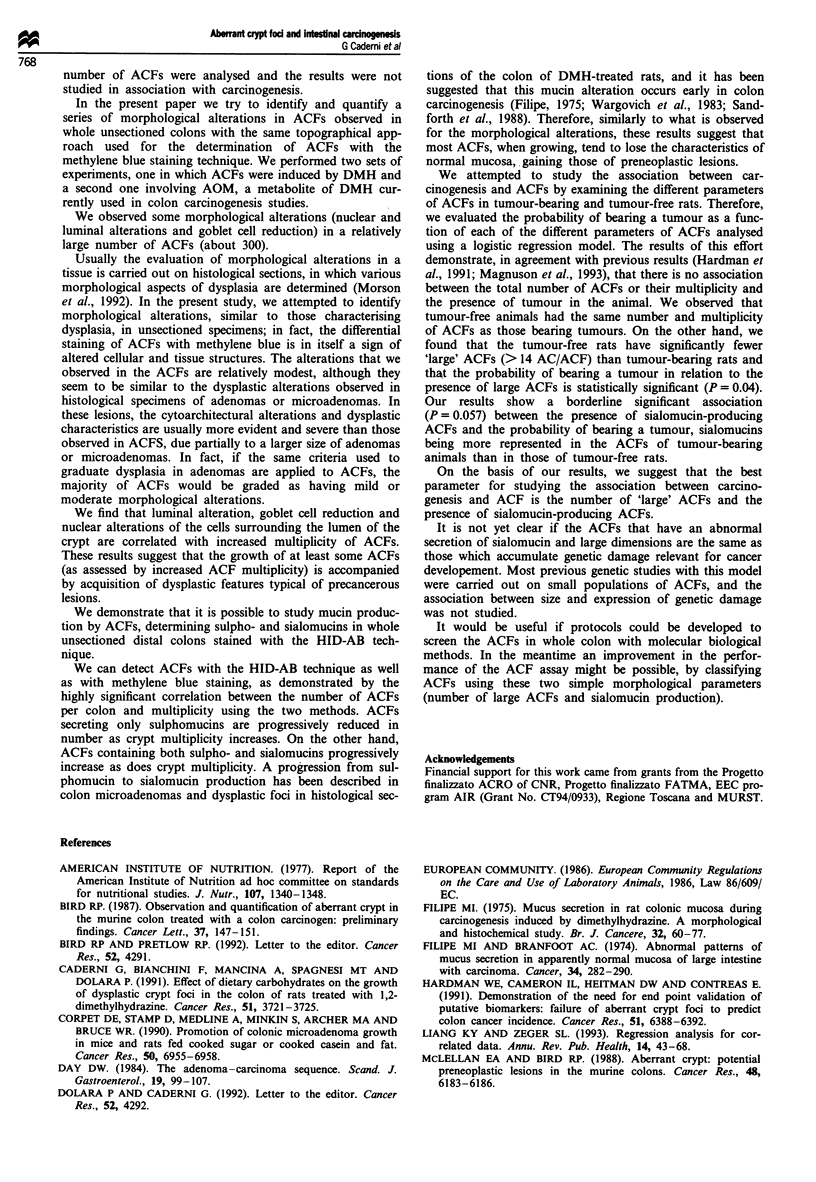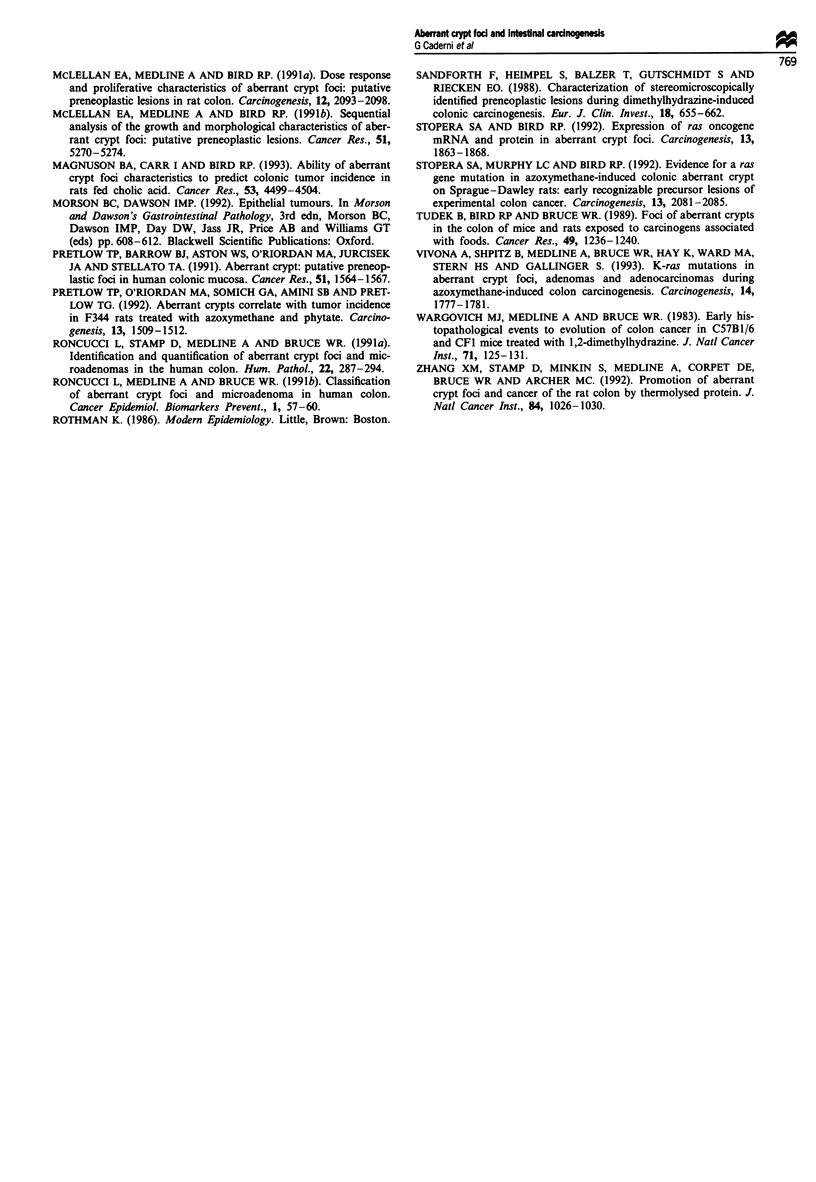# Characterisation of aberrant crypt foci in carcinogen-treated rats: association with intestinal carcinogenesis.

**DOI:** 10.1038/bjc.1995.148

**Published:** 1995-04

**Authors:** G. Caderni, A. Giannini, L. Lancioni, C. Luceri, A. Biggeri, P. Dolara

**Affiliations:** Department of Pharmacology, University of Florence, Italy.

## Abstract

**Images:**


British Journal of Cancer (1995) 71, 763–769
© 1995 Stockton Press All rights reserved 0007-0920/95 $12.00

### Characterisation of aberrant crypt foci in carcinogen-treated rats: association with intestinal carcinogenesis

G Caderni[1], A Giannini[2], L Lancioni[1], C Luceri[1], A Biggeri[3] and P Dolara[1]

[1]*Department of Pharmacology, University of Florence, Viale G.B. Morgagni, 65, 50134 Florence, Italy;* [2]*USL 10/H Antella and Institute of Pathology and* [3]*Department of Statistics, University of Florence, Viale G.B. Morgagni, 59, 50134 Florence, Italy.*

**Summary** Carcinogen-treated rats develop foci of aberrant crypts in the colon (ACFs) that have been interpreted as preneoplastic lesions. To characterise ACFs further, we studied in the unsectioned colon of rats the number, multiplicity, some morphological characteristics and the type of mucin production in ACFs. In ACFs observed 115 days after the administration of 50 mg $kg^{-1}$ 1,2-dimethylhydrazine (DMH), crypt multiplicity [number of aberrant crypts (AC) per focus] was positively correlated ($P < 0.0001$) with the reduction of goblet cells, and with luminal and nuclear alterations in the cells surrounding the lumen of the ACs. We studied mucin production in the unsectioned colon, demonstrating that ACFs producing sulphomucins (like the normal distal rat colon) were progressively reduced when ACF multiplicity increased, whereas ACFs containing sialomucins (correlated with an increased risk of colon cancer) or both sulphomucins and sialomucins increased with crypt multiplicity. We also studied ACFs in the colon and the occurrence of intestinal tumours in rats treated with azoxymethane (AOM; 64 mg $kg^{-1}$). A significant association was found ($P = 0.04$) between tumours and the presence of 'large' ACFs (AC/ACF > 14 crypts) and a borderline significant association ($P = 0.057$) between the presence of tumours and sialomucin-producing ACFs. We found no association between the number of ACFs, ACF multiplicity and the presence of tumours.

**Keywords**: aberrant crypt foci; intestinal carcinogenesis; mucins

Data from experimental animals and humans indicate that colon carcinogenesis is a multistep process in which subsequent preneoplastic lesions accumulate in some mucosa cells, leading finally to neoplastic transformation (Day, 1984; Morson *et al.*, 1992).

Recently, it has been reported that in the colon of rodents treated with specific colon carcinogens, single aberrant crypts (AC) or foci of aberrant crypts (ACFs) can be visualised at low magnification in the unsectioned colon stained with methylene blue (Bird, 1987). ACFs have also been described in the resected colonic mucosa of humans at high risk for colon cancer (Pretlow *et al.*, 1991; Roncucci *et al.*, 1991*a*), where they were interpreted as preneoplastic lesions.

The study of preneoplastic lesions is very important since it allows investigations of the stepwise process leading to cancer. Moreover, preneoplastic lesions can be usesd as end points in studies of experiments carcinogenesis and as early risk indicators for humans. However, since the first description of ACFs in the literature (Bird, 1987), some researchers have wondered whether ACFs may truly be considered preneoplastic lesions and they have tried to identify dysplastic characteristics (such as nuclear alterations and abnormal luminal structure) or molecular abnormalities (such as activation of some oncogenes) in ACFs (McLellan *et al.*, 1991*a,b*; Roncucci *et al.*, 1991*b*; Stopera and Bird, 1992; Stopera *et al.*, 1992; Vivona *et al.*, 1993). The results of these studies indicate that some ACFs are indeed dysplastic lesions. However, only a limited number of ACFs have been evaluated in the previous studies, and it is not yet clear whether all the ACFs that are observed in the methylene blue-stained colon possess dysplastic features. Nor is it clear whether ACFs can be considered a reliable end point in experimental colon carcinogenesis (Hardman *et al.*, 1991; Bird and Pretlow, 1992; Dolara and Caderni, 1992).

In fact, to use ACFs as predictor of cancer frequency in treated animals, different methods have been adopted to define the characteristics of ACFs to be correlated with carcinogenesis. In some studies the total number of ACFs or ACF crypt multiplicity have been used; in some others the numbers of 'large' ACFs were considered (Corpet *et al.*, 1990; Pretlow *et al.*, 1992; Zhang *et al.*, 1992; Magnuson *et al.*, 1993). Although in most studies large ACFs are considered an indication of rapid growth, no consensus has thus far been reached on the most appropriate method of relating ACFs to carcinogenesis.

To characterise further the pathology of ACFs and its correlation with carcinogenesis, we decided to study ACFs in the unsectioned colon of rats at different times after 1,2-dimethylhydrazine (DMH) or azoxymethane (AOM) administration. Accordingly, we determined in ACFs induced by these two carcinogens the number, multiplicity and some morphological alterations such as an abnormal luminal pattern, the reduction in goblet cells and the nuclear alteration in the cells surrounding the aberrant crypts. We were also interested in studying mucin production in ACFs observed in unsectioned colons. Previous investigations performed in histological sections have shown that apparently normal colonic mucosa from patients with colon cancer and dysplastic foci observed in the distal colon of carcinogen-treated rats (Filipe and Branfoot, 1974; Filipe, 1975; Wargovich *et al.*, 1983; Sandforth *et al.*, 1988) produce predominantly sialomucins instead of sulphomucins as the normal mucosa.

### Materials and methods

### *Induction of ACFs by DMH and AOM*

ACFs were induced with two different experimental protocols. In the first we used 8- to 9-week-old female Sprague–Dawley rats (Morini, Italy). Animals were fed a diet based on the AIN 76 diet (American Institute of Nutrition, 1977), modified to contain a relatively high amount of fat (23% corn oil), 23% sucrose and 23% starch as source of carbohydrates (Caderni *et al.*, 1991). The rats were treated twice p.o., 4 days apart, either with saline or with DMH (Sigma Chimica, Milan, Italy) at a dose of 25 mg $kg^{-1}$ body weight (total dose 50 mg $kg^{-1}$). Forty and 115 days after the first treatment with DMH the animals were sacrificed by decapitation under a light ether anaesthesia. Each group was composed of 16 saline-treated and 16 DMH-treated rats.

In the second protocol we used male Sprague–Dawley rats (4–5 weeks old), fed a diet of the same composition as

Correspondence: G Caderni
Received 21 July 1994; revised 31 October 1994; accepted 22 November 1994.

described above. One week after the beginning of the experimental diet rats were treated s.c. with AOM (Sigma Chimica) at a weekly dose of 8 mg kg$^{-1}$ body weight for 8 weeks (total dose 64 mg kg$^{-1}$). We had 60 AOM-treated and 15 saline-treated rats. Between 158 and 162 days after the first dose of AOM the animals were sacrificed by decapitation and ACFs and intestinal tumours were studied. Cancer histological types were evaluated on the basis of histotype, grading and pattern of growth according to Morson *et al.* (1992).

In all the experiments rats were maintained at a constant environmental temperature of 22°C, with a 12 h light–dark cycle, according to internationally accepted ethical guidelines for the treatment of experimental animals (European Community, 1986).

*Determination of the number, multiplicity and morphological alterations of ACFs*

To visualise the ACFs induced by DMH or AOM treatments, we stained the unsectioned colon with methylene blue according to Bird (1987). For each rat we determined the number of ACFs in the entire colon and their multiplicity by counting the number of ACs forming each focus (AC/ACF).

Moreover, in the colon of the rats sacrificed 115 days after DMH, 20 ACFs were randomly chosen from each rat and evaluated for the following architectural and cytological characteristics: pattern of luminal outline of the ACs (referred to as 'luminal alteration'), nuclear alteration with appearance of pseudostratification of the nuclei in the cells surrounding the lumen (referred to as 'nuclear alteration') and reduction in the number of goblet cells. This evaluation was carried out on unsectioned colons stained with methylene blue (magnification 40–100 ×). To evaluate the luminal alteration of the ACs, each AC was graded as mildly altered if the luminal outline was enlarged and round, moderately altered if elliptical or severely altered if slit-like, irregular and cribriform. In the cytological analysis of the nuclear alteration each AC was graded as mildly, moderately or severely altered depending on whether the nuclei, which in normal cells constitute a single layer close to the bases of the cells, seemed to occupy less than 35%, 35–50% or more than 50% of the cell space (epithelium) respectively. In the analysis of the reduction of goblet cells each AC was graded as mildly, moderately or severely altered depending on whether the reduction in the number of goblet cells was, respectively, less than 10%, 10–50% or more than 50% compared with those surrounding the normal crypts. To each grade of morphological alteration we arbitrarily attributed the value of 1, 2 or 3 (for mild, moderate and severe alteration respectively). Finally, the overall grade of ACF alteration was calculated by summing these values for each AC forming the focus and then dividing this value by the number of ACs in that focus.

*Determination of mucus production in the ACFs*

After the determination of the number of multiplicity of the ACFs using the methylene blue staining method, the colons were kept in buffered formalin and later processed with the high-iron diamine Alcian blue (HID-AB) procedure for the analysis of mucus production (Filipe, 1975). Briefly, the entire colon was rinsed for 5 min in distilled water and then transferred into a Petri dish containing a freshly prepared solution, referred to as diamine solution, obtained by dissolving simultaneously 120 mg of *N-N′*-dimethyl-*m*-phenylene diamine and 20 mg of *N-N′*-dimethyl-*p*-phenylene diamine (Sigma Chimica) in 50 ml of distilled water and then adding 1.4 ml of 60% ferric chloride. The Petri dish was covered with aluminium foil for protection against the light and the colon was stained by the diamine solution for 18 h at room temperature. The colon was then rinsed three times in distilled water and stained for 30 min with a solution of 1% Alcian blue (Sigma Chimica) in 3% acetic acid. The colon was then rinsed three times with 80% ethanol followed by distilled water and observed under the microscope with the mucosal side up at a magnification of 40 ×. As a result of this procedure the cells surrounding the opening of the crypts in the distal part of the colon were stained dark brown, indicating a predominance of sulphomucin secretion. Moving from the distal to the proximal part of the colon the cells surrounding the opening of the crypts became increasingly blue, indicating a predominance of sialomucin secretion. This shift from sulphomucin to sialomucin production along the length of the rat colon, going towards the proximal part, has been previously described in histological sections (Filipe, 1975).

We examined only the distal part of the rat colons since the distal colon shows a pattern of mucus production similar to that of the normal human colorectal mucosa in which sulphomucin secretion predominates and since precancerous alterations in the human and distal rat colon are accompanied by a shift from sulphomucin to sialomucin secretion (Filipe and Branfoot, 1974; Filipe, 1975). The examination of DMH-treated rats showed that after the HID-AB procedure the ACFs were stained either dark brown, like the normal surrounding crypts (in which sulphomucin production predominates), or blue (in which sialomucin production predominates; Figure 1a), or both brown and blue together

**Figure 1** Distal colon of a rat 115 days after treatment with DMH, stained with the HID-AB technique as described in Materials and methods. (**a**) A blue ACF secreting sialomucins is surrounded by normal crypts producing sulphomucins (dark brown). (**b**) An ACF producing both sulpho- and sialomucins (blue–brown) is surrounded by normal sulphomucin-producing crypts (magnification 100 ×).

(in which sulphomucins and sialomucins were present together; Figure 1b). The dark-brown ACFs were easily distinguished from the normal crypts, being larger and with a thicker epithelial lining; the blue and blue-brown ACFs, besides the morphological characteristics pertaining to these lesions (Bird, 1987), were easily identified against the background of normal brown crypts (Figure 1a and b). The number of ACFs and their multiplicity (AC/ACF) scored with the methylene blue and with HID-AB technique were significantly correlated ($r = 0.88$ and 0.96 for the number of ACFs and AC/ACF respectively).

### *Statistical analysis of the data*

Differences between means were evaluated using Student's *t*-test for unpaired samples; the correlations between the alterations of the morphological parameters and the multiplicity of ACFs were analysed by a linear regression model using Statgraphics Statistical Package (Statistical Graphic Corporation, Rockville, MD, USA).

To establish whether an ACF parameter would relate to tumour incidence in carcinogen-treated rats, we evaluated the probability of bearing a tumour as a function of each of the different parameters of ACFs analysed, by means of a logistic regression model in which the binary response was bearing or not bearing a tumour. For each parameter, the regression coefficient is an estimate of this association. The odds ratio (OR) of each parameter is calculated as $e^{\text{regression coefficient}} = \text{OR}$; the corresponding 95% confidence limits (CLs) are obtained from the model fitting. The association is considered statistically significant when the 95% CLs do not include the value of 1 (Rothman, 1986).

The association between the distribution of mucin production and bearing or not bearing a tumour was evaluated according to the generalised estimating equation (GEE) (Liang and Zeger, 1986), which is based on a logistic regression model. From this analysis we calculated odds ratios (OR) and the corresponding 95% CLs as described above. The application of GEE is appropriate when there is a correlation among observations. In fact, within the same rat, three measurements were determined: the number of sulphomucin-producing ACFs, the number of ACFs producing sulpho- and sialomucins and the number of ACFs producing sialomucins. These measurements are correlated with each other ($r = 0.45$), in the same rat (cluster). Therefore, the dependence among observations from the same cluster (rat) must be accounted for in assessing the relationship between the different risk factors and tumour outcome.

### Results

### *Characterisation of ACFs 40 and 115 days after DMH administration*

We studied ACFs in the colons of rats treated twice, 4 days apart, with 25 mg $kg^{-1}$ DMH or saline and sacrificed 40 and 115 days after DMH administration. The colon was first stained with methylene blue as described and the number of multiplicity of ACFs were determined. The results indicated that the number of ACFs was similar in the rats sacrificed 40 or 115 days after DMH administration ($82.5 \pm 13.8$ and $85.2 \pm 10.2$, means ± s.e.). As expected, the results also indicated that the multiplicity of ACFs (number of ACs/ACF) was lower in rats sacrificed 40 days after DMH ($1.78 \pm 0.05$) than in rats sacrificed 115 days after ($2.92 \pm 0.17$, means ± s.e., $P < 0.001$). No ACFs were found in the colon of saline-treated rats.

We then evaluated 20 ACFs randomly chosen in each rat sacrificed 115 days after DMH (total number of ACFs analysed = 313 – 312) for the following morphological characteristics: nuclear alteration in the cells surrounding the lumen of the ACs, luminal alteration and reduction in the number of goblet cells as described in Materials and methods. This evaluation was carried out by observing these parameters in the unsectioned colon, since with this topographical approach it was possible to study more lesions than in histological sections.

Each AC or ACF was graded as having mild, moderate or severe alterations as described in the Materials and methods section. The results indicated (Table I) that most ACs had mild or moderate alterations and only a few showed severe nuclear alteration and goblet cell reduction. We also determined the grades of morphological alterations in ACFs as a function of their multiplicity (AC/ACF). The results indicated (Figure 2a–c) that crypt multiplicity was positively correlated with the severity of all the morphological alterations analysed (luminal alteration, goblet cell reduction and nuclear alteration in the cells surrounding the lumen of the crypt).

We also studied the type of mucus produced by the ACFs. The distal colon of each rat treated with DMH and sacrificed 40 and 115 days later was therefore stained with the HID-AB technique and the number and multiplicity of the ACFs and type of mucus secreted were recorded. The results relative to the rats sacrificed 115 days after DMH (Table II) indicated that some of the ACFs still retained the sulphomucin secretion typical of the normal mucosa but that most ACFs secreted both sulpho- and sialomucins. A smaller fraction of ACFs secreted only sialomucins.

Similarly, 40 days after the carcinogen treatment, most ACFs secreted sulpho- and sialomucins (data not shown).

We calculated the correlation between the type of mucin produced by the ACFs and ACF multiplicity. The results relative to ACFs observed 115 days after DMH indicated (Figure 2d) that, when ACF multiplicity increased, the percentage of ACFs producing sulphomucins decreased rapidly, while the percentage of ACFs containing both sulphomucins and sialomucins progressively increased. A few ACFs composed entirely of sialomucins were also observed in ACFs formed by few crypts, and their number slowly increased with the increase in ACF muliplicity. The ACFs observed in rats sacrificed 40 days after DMH showed a similar correlation between mucin production and crypt multiplicity (data not shown).

### *Characterisation of ACFs determined 160 days after AOM administration in rats with and without tumours*

Rats were treated with 8 mg $kg^{-1}$ AOM weekly for 8 weeks and sacrificed 158–162 days after the first injection with AOM. At this time (about 160 days after AOM) we studied intestinal tumours and ACFs in the colon. The results of histopathological examination of the whole intestine (Table III) indicated that the majority of tumours (both adenocarcinomas and adenomas) were induced in the colon, and only a few were found in the small intestine. The control rats treated with saline did not develop tumours.

Since we were interested in studying the ACFs also when tumours were evident, after excision of the tumours for histopathology, the whole colon, fixed in buffered formalin, was stained with methylene blue in order to determine the number and crypt multiplicity of the ACFs. The results of this study indicated that ACFs per colon were $234 \pm 32$ and that their multiplicity (AC/ACF) was $2.53 \pm 0.09$ (means ± s.e.). In the control rats only one animal developed four ACFs, with multiplicity (AC/ACF) = 3. No ACFs were found in the other saline-treated rats.

**Table I** Distribution of different grades of morphological alteration in ACs in the colon of rats 115 days after DMH treatment

| *Parameter* | *Mild* | *Moderate* | *Severe* |
|---|---|---|---|
| Nuclear alteration[a] | 74 (651) | 25.8 (227) | 0.2 (2) |
| Luminal alteration[a] | 37.5 (328) | 62.5 (546) | 0 (0) |
| Goblet cell reduction[a] | 64.6 (567) | 34.5 (303) | 0.9 (8) |

[a]Values represent the percentage of ACs over total ACs in each grade of morphological alteration. The absolute number of ACs is given in parentheses.

**Figure 2** (a–c) Correlation between ACF multiplicity (AC/ACF) and individual values of morphological alteration graded with arbitrary values from 1 to 3 according to progressively severe morphological alterations of the AC forming a focus. The morphological characteristics analysed were (a) luminal alterations (number of ACFs analysed $n = 312$); (b) reduction in the number of goblet cells ($n = 313$); (c) nuclear alterations ($n = 313$). Given the large number of ACFs studied, it was impossible to draw all the experimental points in the figure. Therefore, circles with different diameters have been drawn proportional to the different number of ACFs that they represent. The filled circles represent points for which only one ACF has been found. In each panel ⊕ $n = 50$ gives a graphical representation of this number of observations. (d) Distribution of the ACF multiplicity in the distal colon according to the type of mucus produced by the ACF. For each value of ACF multiplicity (AC/ACF) we calculated the percentage of ACF/total ACFs secreting sulphomucins (-●-), sulphomucins and sialomucins together (-■-) and sialomucins (-▲-).

**Table II** Mucin production[a] in ACFs observed in the colon of rats 115 days after DMH treatment

| *Sulphomucins* | *Sulpho-sialomucins* | *Sialomucins* |
|---|---|---|
| 26.6 (363) | 58.4 (796) | 15 (205) |

[a]Values represent the percentage of ACFs over total ACFs in each category of mucus secretion. The absolute number of ACFs counted is given in parentheses.

**Table III** Tumour outcome in rats treated with multiple injections of AOM (total dose 64 mg $kg^{-1}$)

| | |
|---|---|
| Colonic adenocarcinomas per rat[a] | 0.55 ± 0.11 |
| Colonic adenomas per rat[a] | 0.59 ± 0.15 |
| Small intestinal adenocarcinomas per rat[a] | 0.11 ± 0.04 |
| Small intestinal adenomas per rat[a] | 0.02 ± 0.02 |
| Frequency of total intestinal tumours[b] | 64.6% |

[a]The values are the mean number of each type of tumour per rat ± s.e., $n = 54$. [b]The value represents the percentage of rats with intestinal (colonic and small intestinal) tumours (adenomas and adenocarcinomas).

The distal part of the colons was also stained with the HID-AB technique to determine mucin production. We found that the distribution of ACFs secreting different types of mucins was similar to that observed in the DMH experiments. In fact, we found that 34% of ACFs secreted sulphomucins, 55.3% a mixture of sulpho- and sialomucins and 10.4% of ACFs secreted only sialomucins.

Since we wanted to evaluate which characteristics of ACFs would related to tumour occurrence in the treated rats, we determined the number and multiplicity of the ACFs and the type of mucin produced in tumour-bearing and tumour-free rats. We also examined whether the probability of bearing a tumour was associated with each of the different ACF parameters analysed, calculating odds ratios (OR) as a measure of this association and the corresponding 95% CLs with a logistic regression model as explained in detail in the Materials and methods section.

As illustrated in Figure 3a, the animals which developed tumours tended to have more ACFs than tumour-free rats; analysis of these results with the logistic model indicated that the probability of developing tumours is not statistically associated with the number of ACFs per colon (OR = 1.003, 95% CLs = 0.996–1.008). Crypt multiplicity (Figure 3b) was similar in rats with or without tumours (OR = 0.57, 95% CLs = 0.12–2.64).

Since it has been suggested that the number of 'large' ACFs could be used to predict colon carcinogenesis in

**Figure 3** Some ACF parameters in tumour-bearing (■) and in tumour-free rats (□). (**a**) Number of ACFs per colon. (**b**) ACF multiplicity (AC/ACF). (**c**) Number of ACFs per colon equal to or larger than 14 AC/ACF. $*P<0.05$ compared with the tumour-bearing rats. Values are means ± s.e.; $n = 26$ and 12 in tumour-bearing and tumour-free rats respectively.

rodents treated with carcinogens (Corpet *et al*., 1990; Pretlow *et al*., 1992; Zhang *et al*., 1992), we also evaluated this parameter in tumour-bearing and tumour-free rats. We defined 'large' ACFs according to Corpet *et al*. (1990) as being of such a size that at least one large ACF per rat was present. Using this criterion, 'large' ACFs in our AOM experiment were those formed by 14 or more crypts. The results obtained (Figure 3c) showed that the number of large ACFs in the rats which developed tumours was significantly higher than in tumour-free rats; the analysis relative to the probability of bearing a tumour as a function of the presence of a high number of large ACFs showed a significant association between the presence of a tumour and number of large ACFs: OR = 3.30 with 95% CLs = 1.1–10.2. On the other hand, defining 'large ACFs' according to Zhang *et al*. (1992) (in our AOM experiment ACFs formed by ten or more crypts), the animals with tumours had a higher number of large ACFs than tumour-free rats, but the difference was not statistically significant (large ACFs were 3.38 ± 0.75 and 1.87 ± 0.35 in the tumour-bearing and tumour-free rats respectively; means ± s.e.).

**Table IV** Distribution of ACFs secreting different types of mucins in AOM-treated rats both with tumours and tumour-free

| | *Sulphomucins* | *Sulpho-sialomucins* | *Sialomucins* |
|---|---|---|---|
| *Tumour-bearing rats* | 34.7 (931) | 53.2 (1430) | 12.1 (325) |
| *Tumour-free rats* | 33.2 (397) | 60 (717) | 6.7 (80)[a] |

Values represent the percentage of ACFs over total ACFs. The absolute number of ACFs found in each category of mucus secretion is given in parentheses. [a]The differences between the percentage of sialomucin-producing ACFs in tumour-bearing and tumour-free rats is borderline statistically significant ($P = 0.057$) as determined by generalised estimating equation analysis (see the Materials and methods section for explanation).

We also evaluated the type of mucin produced by the ACFs in tumour-bearing and tumour-free rats. The distribution of the different types of mucin produced by the ACFs in tumour-bearing and in tumour-free rats (Table IV) showed that in tumour-free rats fewer sialomucins and more mixed sulpho- and sialomucins were present. The results indicated a borderline significant association ($P = 0.057$) between the presence of sialomucin-producing ACFs and the probability of bearing a tumour (OR = 2.04 with 95% CLs 1–4.2).

### Discussion

In recent years several laboratories have been using ACFs as an end point in short-term tests for predicting colon carcinogenesis in rodents (Tudek *et al*., 1989; Corpet *et al*., 1990; Pretlow *et al*., 1990; Zhang *et al*., 1992). ACFs are easily scored in the unsectioned colon of carcinogen-treated animals, and they develop as early as 2–4 weeks after carcinogen administration. Moreover, the ACF assay requires a relatively small number of animals and a short experimental time as compared with long-term carcinogenesis studies. Therefore this assay has attained considerable popularity as a short-term test for the study of the modulating effects of chemicals and/or dietary factors on experimental colon carcinogenesis.

Notwithstanding the merits of the ACF assay, the relationship between ACFs and carcinogenesis seems to be rather complicated. It is certain that colon carcinogens induce ACFs dose dependently and that the number of ACFs is correlated with the potency of the carcinogen (Tudek *et al*., 1993). However, some authors (Hardman *et al*., 1991) have not observed an association between the incidence of colon cancer and ACF number or size, and most others do not use the same parameters to describe the association of ACFs with carcinogenesis. Some authors stress the importance of ACF multiplicity (AC/ACF) (Magnuson *et al*., 1993), while others favour the number of 'large' ACFs, but they calculate 'large' ACFs using different criteria (Corpet *et al*., 1990; Pretlow *et al*., 1992; Zhang *et al*., 1992).

Given the current undetermined relationship between ACFs and carcinogenesis, the validity of the ACF assay has been questioned (Hardman *et al*., 1991; Magnuson *et al*., 1993) and the possibility has been raised that ACFs are elementary lesions, only a few of which are truly preneoplastic.

Some authors have also studied genetic lesions which occur in colon adenomas and carcinomas in ACFs. Accordingly, it has been demonstrated that a small subpopulation of ACFs have increased expression and activation of the *ras* oncogene (Stopera and Bird, 1992; Stopera *et al*., 1992; Vivona *et al*., 1993). Other studies have focused on ACF dysplasia, with the aim of identifying the actual dysplastic lesions within the ACF population (McLellan *et al*., 1991*b*; Roncucci *et al*., 1991*b*). The limitation of these studies is that only a small number of ACFs were analysed and the results were not studied in association with carcinogenesis.

In the present paper we try to identify and quantify a series of morphological alterations in ACFs observed in whole unsectioned colons with the same topographical approach used for the determination of ACFs with the methylene blue staining technique. We performed two sets of experiments, one in which ACFs were induced by DMH and a second one involving AOM, a metabolite of DMH currently used in colon carcinogenesis studies.

We observed some morphological alterations (nuclear and luminal alterations and goblet cell reduction) in a relatively large number of ACFs (about 300).

Usually the evaluation of morphological alterations in a tissue is carried out on histological sections, in which various morphological aspects of dysplasia are determined (Morson *et al.*, 1992). In the present study, we attempted to identify morphological alterations, similar to those characterising dysplasia, in unsectioned specimens; in fact, the differential staining of ACFs with methylene blue is in itself a sign of altered cellular and tissue structures. The alterations that we observed in the ACFs are relatively modest, although they seem to be similar to the dysplastic alterations observed in histological specimens of adenomas or microadenomas. In these lesions, the cytoarchitectural alterations and dysplastic characteristics are usually more evident and severe than those observed in ACFS, due partially to a larger size of adenomas or microadenomas. In fact, if the same criteria used to graduate dysplasia in adenomas are applied to ACFs, the majority of ACFs would be graded as having mild or moderate morphological alterations.

We find that luminal alteration, goblet cell reduction and nuclear alterations of the cells surrounding the lumen of the crypt are correlated with increased multiplicity of ACFs. These results suggest that the growth of at least some ACFs (as assessed by increased ACF multiplicity) is accompanied by acquisition of dysplastic features typical of precancerous lesions.

We demonstrate that it is possible to study mucin production by ACFs, determining sulpho- and sialomucins in whole unsectioned distal colons stained with the HID-AB technique.

We can detect ACFs with the HID-AB technique as well as with methylene blue staining, as demonstrated by the highly significant correlation between the number of ACFs per colon and multiplicity using the two methods. ACFs secreting only sulphomucins are progressively reduced in number as crypt multiplicity increases. On the other hand, ACFs containing both sulpho- and sialomucins progressively increase as does crypt multiplicity. A progression from sulphomucin to sialomucin production has been described in colon microadenomas and dysplastic foci in histological sections of the colon of DMH-treated rats, and it has been suggested that this mucin alteration occurs early in colon carcinogenesis (Filipe, 1975; Wargovich *et al.*, 1983; Sandforth *et al.*, 1988). Therefore, similarly to what is observed for the morphological alterations, these results suggest that most ACFs, when growing, tend to lose the characteristics of normal mucosa, gaining those of preneoplastic lesions.

We attempted to study the association between carcinogenesis and ACFs by examining the different parameters of ACFs in tumour-bearing and tumour-free rats. Therefore, we evaluated the probability of bearing a tumour as a function of each of the different parameters of ACFs analysed using a logistic regression model. The results of this effort demonstrate, in agreement with previous results (Hardman *et al.*, 1991; Magnuson *et al.*, 1993), that there is no association between the total number of ACFs or their multiplicity and the presence of tumour in the animal. We observed that tumour-free animals had the same number and multiplicity of ACFs as those bearing tumours. On the other hand, we found that the tumour-free rats have significantly fewer 'large' ACFs (>14 AC/ACF) than tumour-bearing rats and that the probability of bearing a tumour in relation to the presence of large ACFs is statistically significant ($P = 0.04$). Our results show a borderline significant association ($P = 0.057$) between the presence of sialomucin-producing ACFs and the probability of bearing a tumour, sialomucins being more represented in the ACFs of tumour-bearing animals than in those of tumour-free rats.

On the basis of our results, we suggest that the best parameter for studying the association between carcinogenesis and ACF is the number of 'large' ACFs and the presence of sialomucin-producing ACFs.

It is not yet clear if the ACFs that have an abnormal secretion of sialomucin and large dimensions are the same as those which accumulate genetic damage relevant for cancer developement. Most previous genetic studies with this model were carried out on small populations of ACFs, and the association between size and expression of genetic damage was not studied.

It would be useful if protocols could be developed to screen the ACFs in whole colon with molecular biological methods. In the meantime an improvement in the performance of the ACF assay might be possible, by classifying ACFs using these two simple morphological parameters (number of large ACFs and sialomucin production).

**Acknowledgements**

Financial support for this work came from grants from the Progetto finalizzato ACRO of CNR, Progetto finalizzato FATMA, EEC program AIR (Grant No. CT94/0933), Regione Toscana and MURST.

**References**

AMERICAN INSTITUTE OF NUTRITION. (1977). Report of the American Institute of Nutrition ad hoc committee on standards for nutritional studies. *J. Nutr.*, **107**, 1340–1348.

BIRD RP. (1987). Observation and quantification of aberrant crypt in the murine colon treated with a colon carcinogen: preliminary findings. *Cancer Lett.*, **37**, 147–151.

BIRD RP AND PRETLOW RP. (1992). Letter to the editor. *Cancer Res.*, **52**, 4291.

CADERNI G, BIANCHINI F, MANCINA A, SPAGNESI MT AND DOLARA P. (1991). Effect of dietary carbohydrates on the growth of dysplastic crypt foci in the colon of rats treated with 1,2-dimethylhydrazine. *Cancer Res.*, **51**, 3721–3725.

CORPET DE, STAMP D, MEDLINE A, MINKIN S, ARCHER MA AND BRUCE WR. (1990). Promotion of colonic microadenoma growth in mice and rats fed cooked sugar or cooked casein and fat. *Cancer Res.*, **50**, 6955–6958.

DAY DW. (1984). The adenoma–carcinoma sequence. *Scand. J. Gastroenterol.*, **19**, 99–107.

DOLARA P AND CADERNI G. (1992). Letter to the editor. *Cancer Res.*, **52**, 4292.

EUROPEAN COMMUNITY. (1986). *European Community Regulations on the Care and Use of Laboratory Animals*, 1986, Law 86/609/EC.

FILIPE MI. (1975). Mucus secretion in rat colonic mucosa during carcinogenesis induced by dimethylhydrazine. A morphological and histochemical study. *Br. J. Cancere*, **32**, 60–77.

FILIPE MI AND BRANFOOT AC. (1974). Abnormal patterns of mucus secretion in apparently normal mucosa of large intestine with carcinoma. *Cancer*, **34**, 282–290.

HARDMAN WE, CAMERON IL, HEITMAN DW AND CONTREAS E. (1991). Demonstration of the need for end point validation of putative biomarkers: failure of aberrant crypt foci to predict colon cancer incidence. *Cancer Res.*, **51**, 6388–6392.

LIANG KY AND ZEGER SL. (1993). Regression analysis for correlated data. *Annu. Rev. Pub. Health*, **14**, 43–68.

MCLELLAN EA AND BIRD RP. (1988). Aberrant crypt: potential preneoplastic lesions in the murine colons. *Cancer Res.*, **48**, 6183–6186.

McLELLAN EA, MEDLINE A AND BIRD RP. (1991*a*). Dose response and proliferative characteristics of aberrant crypt foci: putative preneoplastic lesions in rat colon. *Carcinogenesis*, **12**, 2093–2098.

McLELLAN EA, MEDLINE A AND BIRD RP. (1991*b*). Sequential analysis of the growth and morphological characteristics of aberrant crypt foci: putative preneoplastic lesions. *Cancer Res.*, **51**, 5270–5274.

MAGNUSON BA, CARR I AND BIRD RP. (1993). Ability of aberrant crypt foci characteristics to predict colonic tumor incidence in rats fed cholic acid. *Cancer Res.*, **53**, 4499–4504.

MORSON BC, DAWSON IMP. (1992). Epithelial tumours. In *Morson and Dawson's Gastrointestinal Pathology*, 3rd edn, Morson BC, Dawson IMP, Day DW, Jass JR, Price AB and Williams GT (eds) pp. 608–612. Blackwell Scientific Publications: Oxford.

PRETLOW TP, BARROW BJ, ASTON WS, O'RIORDAN MA, JURCISEK JA AND STELLATO TA. (1991). Aberrant crypt: putative preneoplastic foci in human colonic mucosa. *Cancer Res.*, **51**, 1564–1567.

PRETLOW TP, O'RIORDAN MA, SOMICH GA, AMINI SB AND PRETLOW TG. (1992). Aberrant crypts correlate with tumor incidence in F344 rats treated with azoxymethane and phytate. *Carcinogenesis*, **13**, 1509–1512.

RONCUCCI L, STAMP D, MEDLINE A AND BRUCE WR. (1991*a*). Identification and quantification of aberrant crypt foci and microadenomas in the human colon. *Hum. Pathol.*, **22**, 287–294.

RONCUCCI L, MEDLINE A AND BRUCE WR. (1991*b*). Classification of aberrant crypt foci and microadenoma in human colon. *Cancer Epidemiol. Biomarkers Prevent.*, **1**, 57–60.

ROTHMAN K. (1986). *Modern Epidemiology*. Little, Brown: Boston.

SANDFORTH F, HEIMPEL S, BALZER T, GUTSCHMIDT S AND RIECKEN EO. (1988). Characterization of stereomicroscopically identified preneoplastic lesions during dimethylhydrazine-induced colonic carcinogenesis. *Eur. J. Clin. Invest.*, **18**, 655–662.

STOPERA SA AND BIRD RP. (1992). Expression of *ras* oncogene mRNA and protein in aberrant crypt foci. *Carcinogenesis*, **13**, 1863–1868.

STOPERA SA, MURPHY LC AND BIRD RP. (1992). Evidence for a *ras* gene mutation in azoxymethane-induced colonic aberrant crypt on Sprague–Dawley rats: early recognizable precursor lesions of experimental colon cancer. *Carcinogenesis*, **13**, 2081–2085.

TUDEK B, BIRD RP AND BRUCE WR. (1989). Foci of aberrant crypts in the colon of mice and rats exposed to carcinogens associated with foods. *Cancer Res.*, **49**, 1236–1240.

VIVONA A, SHPITZ B, MEDLINE A, BRUCE WR, HAY K, WARD MA, STERN HS AND GALLINGER S. (1993). K-*ras* mutations in aberrant crypt foci, adenomas and adenocarcinomas during azoxymethane-induced colon carcinogenesis. *Carcinogenesis*, **14**, 1777–1781.

WARGOVICH MJ, MEDLINE A AND BRUCE WR. (1983). Early histopathological events to evolution of colon cancer in C57Bl/6 and CF1 mice treated with 1,2-dimethylhydrazine. *J. Natl Cancer Inst.*, **71**, 125–131.

ZHANG XM, STAMP D, MINKIN S, MEDLINE A, CORPET DE, BRUCE WR AND ARCHER MC. (1992). Promotion of aberrant crypt foci and cancer of the rat colon by thermolysed protein. *J. Natl Cancer Inst.*, **84**, 1026–1030.